# Chemogenetic Enhancement of cAMP Signaling Renders Hippocampal Synaptic Plasticity Resilient to the Impact of Acute Sleep Deprivation

**DOI:** 10.1523/ENEURO.0380-22.2022

**Published:** 2023-01-03

**Authors:** Emily Nicole Walsh, Mahesh Shivarama Shetty, Kamran Diba, Ted Abel

**Affiliations:** 1 Department of Neuroscience and Pharmacology, Carver College of Medicine, University of Iowa, Iowa City, IA 52242; 2 Iowa Neuroscience Institute, Carver College of Medicine, University of Iowa, Iowa City, IA 52242; 3 Interdisciplinary Graduate Program in Neuroscience, University of Iowa, Iowa City, IA 52242; 4 Department of Anesthesiology, University of Michigan Medical School, Ann Arbor, MI 48109

**Keywords:** cAMP, chemogenetics, hippocampus, long-term potentiation, sleep deprivation, synaptic plasticity

## Abstract

Sleep facilitates memory storage and even brief periods of sleep loss lead to impairments in memory, particularly memories that are hippocampus dependent. In previous studies, we have shown that the deficit in memory seen after sleep loss is accompanied by deficits in synaptic plasticity. Our previous work has also found that sleep deprivation (SD) is associated with reduced levels of cyclic adenosine monophosphate (cAMP) in the hippocampus and that the reduction of cAMP mediates the diminished memory observed in sleep-deprived animals. Based on these findings, we hypothesized that cAMP acts as a mediator for not only the cognitive deficits caused by sleep deprivation, but also the observed deficits in synaptic plasticity. In this study, we expressed the heterologous *Drosophila melanogaster* Gαs-protein-coupled octopamine receptor (DmOctβ1R) in mouse hippocampal neurons. This receptor is selectively activated by the systemically injected ligand (octopamine), thus allowing us to increase cAMP levels in hippocampal neurons during a 5-h sleep deprivation period. Our results show that chemogenetic enhancement of cAMP during the period of sleep deprivation prevents deficits in a persistent form of long-term potentiation (LTP) that is induced at the Schaffer collateral synapses in the hippocampal CA1 region. We also found that elevating cAMP levels in either the first or second half of sleep deprivation successfully prevented LTP deficits. These findings reveal that cAMP-dependent signaling pathways are key mediators of sleep deprivation at the synaptic level. Targeting these pathways could be useful in designing strategies to prevent the impact of sleep loss.

## Significance Statement

Insufficient sleep is an issue with significant health and socioeconomic implications. This includes a negative impact on memory consolidation. Previous studies in mice found that acute sleep deprivation (SD) leads to deficits in hippocampal synaptic plasticity and memory, and those deficits are associated with reduced levels of the signaling molecule cAMP. In this study, we used a chemogenetic strategy to enhance cAMP levels in specific hippocampal neurons during sleep deprivation. We found that this made synaptic plasticity resilient to the negative effects of sleep deprivation. These findings reveal that cAMP-dependent signaling pathways are key mediators of sleep deprivation and that targeting these pathways could be useful in designing strategies to prevent the impact of sleep loss.

## Introduction

It has been known for many years that sleep plays an important role in long-term memory consolidation. Even short periods of sleep can improve declarative memory (Tucker et al., 2006; Van Der Helm et al., 2011), and loss of sleep leads to impairments in memory in both rodents and humans (Graves et al., 2003; Palchykova et al., 2006; Rasch and Born, 2013). One way that sleep is believed to carry out this function is by altering the synaptic plasticity that underlies memory consolidation (Yang and Gan, 2012; Yang et al., 2014; Li et al., 2017). This is particularly true in the hippocampus, an important brain region for the consolidation of episodic memories, which undergoes changes in molecular and cellular signaling and demonstrates impaired synaptic plasticity in response to sleep deprivation (SD). A single, brief period of SD by gentle handling alters hippocampal gene transcription (Vecsey et al., 2012), impairs hippocampal protein synthesis (Tudor et al., 2016; Lyons et al., 2020), and decreases hippocampal cAMP signaling, the latter of which leads to reduced activation of downstream effectors such as cAMP response element-binding protein (CREB; Vecsey et al., 2009), LIM kinase (LIMK), and cofilin (Havekes et al., 2016b). The alterations in molecular signaling caused by SD lead to selective deficits in persistent forms of long-term potentiation (LTP) at the Schaffer collateral synapses in the stratum radiatum of hippocampal area CA1 (Vecsey et al., 2009, 2018; Wong et al., 2019).

The reduction in cAMP levels has been attributed, at least in part, to an increase in the protein levels of the cAMP-specific phosphodiesterase PDE4A5 (Vecsey et al., 2009). Previous work has also shown that the deficits in persistent forms of LTP that are caused by sleep loss could be rescued by treating hippocampal slices with a PDE4 inhibitor to prevent cAMP degradation (Vecsey et al., 2009). A similar strategy of inhibiting PDEs to prevent cAMP degradation also prevents the hippocampal memory deficits that are associated with acute SD (Vecsey et al., 2009; Heckman et al., 2020). However, these studies lacked the spatial or temporal specificity to determine whether cAMP elevation during the SD period could overcome those deficits in memory and LTP. Here, we use a chemogenetic approach to express the heterologous *Drosophila melanogaster* Gαs-protein-coupled octopamine receptor (DmOctβ1R) selectively in excitatory neurons of the hippocampus. When the DmOctβ1R is activated by the delivery of its ligand (octopamine) it elevates cAMP levels in the tissue expressing the receptor (Balfanz et al., 2005; Isiegas et al., 2008; Havekes et al., 2014). This DmOctβ1R approach has previously been used in our lab to elevate cAMP levels during SD, where we demonstrated that this elevation makes animals resilient to the memory deficits that are normally caused by sleep loss (Havekes et al., 2014). In this study, we build on these important experiments to investigate whether elevating hippocampal cAMP levels during SD would also provide resilience against deficits in synaptic plasticity. Using the same approach of virus-mediated expression of the DmOctβ1R in excitatory hippocampal neurons, we show that activation of DmOctβ1R during SD prevents the deficit in long-lasting LTP that is normally caused by sleep deprivation.

## Materials and Methods

### Subjects

Male C57BL/6J mice (The Jackson Laboratory #000664), three to four months of age, were used for all the experiments. Before the start of the experiment, mice were group housed (up to five per cage) in soft bedding cages. Food (NIH-31 irradiated modified mouse diet #7913) and water were provided *ad libitum*. These mice were on a 12/12 h light/dark schedule, lights-on at 8 A.M. (9 A.M. during DST). The start of the lights-on period marks zeitgeber time (ZT) 0. For mice that underwent surgery, the surgery was performed between 10 and 12 weeks of age. Mice were maintained in group housing during the recovery from surgery. Experiments were conducted according to National Institutes of Health *Guidelines for Animal Care* and use and were approved by the Institutional Animal Care and Use Committee (IACUC) at the University of Iowa.

### Viral vectors and surgeries

To manipulate cAMP levels in hippocampal neurons we used an AAV construct (serotype 9; AAV9-CaMKIIα0.4-DmOctβ1R-HA-rBG [titer 1.24E + 14 genome copies (GC)/ml]) containing the *D. melanogaster* octopamine receptor type 1β (DmOctβ1R; Balfanz et al., 2005), which when bound by the octopamine ligand, activates adenylyl cyclase and stimulates cAMP production. The plasmid was generated using Geneart and packaged by the University of Pennsylvania Viral Vector Core. The construct is expressed under the CaMKIIα promoter (0.4-kb fragment) and also contains an HA tag to facilitate visualizing the expression of the receptor. The stock virus was diluted to a lower titer (1.24E + 13 GC/ml) in saline solution (0.9% sodium chloride, Hospira Inc.) before infusion into the hippocampus. To assess the effects of octopamine in the absence of the receptor, an enhanced green fluorescent protein (eGFP) under the CaMKIIα promoter was used (AAV9-CaMKIIα-eGFP-WPRE-rBG; Addgene #50469, 1.2E+13 GC/ml). We infused the AAV into the dorsal hippocampus using the following coordinates relative to bregma: anteroposterior (AP) −1.9 mm, mediolateral (ML) ±1.5 mm, dorsoventral (DV) −1.5 mm. Mice were induced and maintained anaesthetized with isoflurane for the surgery. The AAV suspension was infused bilaterally (1000 nl per hemisphere at a rate of 200 nl/min) using a NanoFil syringe (World Precision Instruments, NanoFil 10 μl) through a 33-G beveled needle (World Precision Instruments, #NF33BV-2), controlled by a microsyringe pump (World Precision Instruments, Microinjection Syringe Pump, #UMP3T-2). We allowed four weeks to pass between the initial virus delivery and the day of the experiment to allow for the virus to express.

### Drug preparation

One milligram of (±)-Octopamine hydrochloride (Millipore Sigma #O0250-5G) was dissolved in 2 ml of 0.9% saline solution, to obtain 0.5 mg/ml concentration of octopamine solution. This solution was prepared fresh on the day of the experiment. The volume was administered based on the weight of the mouse at a dose of 1 mg/kg. For vehicle controls, the appropriate volume of 0.9% saline was used for injections. Octopamine solution or saline was administered to mice by intraperitoneal injection.

### Experimental design

#### Sleep deprivation

All mice were singly housed 7 days before the sleep deprivation (SD) or non-sleep deprivation (NSD) day. Each cage had corncob bedding (Envigo, Teklad ¼” corncob, #7907), and a small amount of soft bedding for mice to make an adequate nest. These cages were equipped with water bottles and wire hoppers to hold food. Mice had *ad libitum* access to food and water at all times, including during SD. Mice were handled for 5 d before the experiment by the same researcher conducting the experiments. This allowed the mice to habituate to the experimenter, room, and the tapping stimulation that was used on the cage to keep mice in the SD group awake. For handling, the mice were taken to the room that would be used for sleep deprivation, and each mouse was held in the experimenter’s palm for 2 min. They were then placed back in their cages, and cages were tapped for 2 min to habituate them to the stimuli that would be used in the gentle handling method of sleep deprivation. For mice in an experiment requiring injection, injection habituation started 2–3 d before the day of SD (0.9% saline vehicle, 0.1 ml, i.p.). The injection was administered after handling, and before mice being returned to their cage for 2 min of tapping. SD began at ZT0 and continued for 5 h using the gentle handling method (Vecsey et al., 2009; Hagewoud et al., 2010; Prince et al., 2014) in which the experimenter taps the side of the cage as needed to keep mice awake. When taps were no longer sufficient the mice received a “cage shake,” which was a motion of the cage to offset the balance of the mouse and rouse them. Mice in the NSD group were housed and handled identically, but on the day of the experiment were instead kept in their behavioral colony housing room throughout the 5-h period.

#### Slice electrophysiology

Immediately after the SD or NSD period (at the end of ZT5), mice were cervically dislocated and the hippocampi were rapidly dissected in artificial CSF (aCSF; 124 mm NaCl, 4.4 mm KCl, 1.3 mm MgSO_4_.7H_2_O, 2.5 mm CaCl_2_.2H_2_O, 1 mm NaH_2_PO_4_.H_2_O, 26.2 mm NaHCO_3_, and 10 mm D-glucose, pH ∼7.4, osmolarity ∼300 mOsm) with continuous flow of carbogen (95% oxygen, 5% carbon dioxide). 400 μm-thick transverse hippocampal slices were prepared from the dorsal 2/3 portion of both the hippocampi by a manual McIlwain slicer (Stoelting), as previously described (Shetty et al., 2015). The slices were placed in a netted interface chamber (Fine Science Tools) and incubated at 28°C for at least 2 h in oxygenated aCSF (perfused at 1 ml/min) before starting electrophysiological recordings. For all recordings, a monopolar, lacquer coated stainless-steel electrode (A-M Systems #571000) was positioned in the CA1 stratum radiatum to stimulate Schaffer collaterals, and an aCSF-filled glass electrode (2 to 5 MΩ resistance) was also placed in the CA1 stratum radiatum to record field EPSPs (fEPSPs). For test stimulation, a biphasic, constant current pulse (100 μs duration per phase) was delivered using an isolated pulse stimulator (Model 2100, A-M Systems) and recorded using IE250 Intracellular Electrometer (Warner Instruments). Data were low-pass filtered at 2 kHz (LPF100B, Warner Instruments) and acquired at 20 kHz using pClamp 10 software and Axon Digidata 1440/1550 digitizers (Molecular Devices). For every slice, an input-output curve (stimulation intensity vs fEPSP amplitude) was generated, and the baseline stimulation intensity was set to elicit ∼40% of the maximal fEPSP amplitude. In all the experiments, test stimulation was performed once every minute, including for 20 min to establish a stable baseline before long-term potentiation (LTP) induction. LTP was induced with a spaced four-train stimulation paradigm (four 100 Hz, 1 s trains delivered 5 min apart, at the baseline intensity) and recordings were continued for 160 min. The data were analyzed using Clampfit 10 analysis software (Axon Molecular Devices). In every experiment, the fEPSP initial slopes (20–80%) were normalized to the 20 min baseline average and expressed as percentages. Input–output characteristics were assessed by quantifying fEPSP and presynaptic fiber volley (PFV) amplitudes in response to increasing stimulus intensity (0–70 μA, at 5 μA increments). Paired-pulse facilitation (PPF) was assessed by delivering two pulses at baseline intensity at different interpulse intervals (300, 200, 100, 50, and 25 ms). Facilitation was quantified by the ratio of the second fEPSP amplitude to the first. In all the electrophysiology experiments, reported *n* values refer to the number of mice, and data from replicate slices from the same mouse are averaged. Mean and SEM are reported in figure legends and Results.

#### Immunohistochemistry (IHC)

To confirm the viral expression of DmOctβ1R-HA in hippocampal tissue, we used an anti-HA IHC protocol with chromogenic 3,3′-diaminobenzidine (DAB) staining. Mice used for IHC staining were perfused with 1× PBS (∼10 ml), followed by 4% paraformaldehyde (PFA; ∼10 ml). Brains were extracted into 4% PFA solution and left overnight (4°C) before being transferred to 30% sucrose solution (4°C). Once equilibrated, the brains were sliced into 30 μm-thick sections on a cryostat (Leica 3050S). All steps for DAB staining were done with gentle rotation and at room temperature unless otherwise stated. First, slices were washed in 1× PBS three times for 5 min each, then incubated for 25 min in H_2_O_2_ (0.3% H_2_O_2_ in 1× PBS). Next, the slices were washed with 1× PBS for 30 min before being preincubated with 5% normal goat serum and 0.1% Triton X-100 in 1× PBS. Following preincubation, slices were incubated overnight with the primary antibody (HA-Tag [C29F4], Cell Signaling #3724S) 1:100 in 1× PBS plus 0.1% Triton X-100 and 1% normal goat serum. The next day, slices were washed for 3 × 10 min with 1× PBS before being incubated for 5 h with the secondary antibody (Biotinylated, Vector Laboratories, catalog #BA-1000) 1:500 in 1× PBS and 1% normal goat serum. Afterwards, they were washed 3 × 10 min with 1× PBS, then incubated for 2 h with ABC kit (VECTASTAIN Elite ABC HRP kit [peroxidase, Standard], catalog #K-6100; 1:100 of both components in 1× PBS). The slices were then washed for 4 h in 1× PBS (one 15-min wash in 1× PBS, then again with fresh 1× PBS every 1–1.5 h for a total of four washes), before being moved into the DAB (DAB-HCl, Electron Microscopy Sciences, Fisher Scientific catalog #50-980-352) solution (0.15 mg DAB/ml of 1× PBS, add 100 μl 0.1% H_2_O_2_ to every 5 ml of the solution right before starting the stain). Slices are incubated in the DAB solution for 8 min. After the DAB step, the slices were washed 3 × 10 min in 1× PBS to arrest further reaction. Slices were moved out of 1× PBS onto Superfrost Plus (Fisherbrand) slides, allowed to dry overnight, and then coverslipped with Permount (Fisher Chemical Permount Mounting Medium, Fisher Scientific #SP15-100). Slices were imaged on the Leica TCS SPE microscope under brightfield image acquisition with a 10× objective. Scale bars for each image are described in the figure legend.

### Statistical analysis

Power analyses were performed for all experiments at an α of 0.05 and a desired power level of 0.80 to estimate the number of mice needed. The estimated effect sizes were based on previous publications using similar methods. All the surgeries and sleep deprivation were performed by one experimenter and the electrophysiology experiments were performed by another experimenter blind to the identity of the mice or condition. Experiments from respective control and experimental mice were performed side-by-side on any given day. In the LTP experiments, the average fEPSP slopes over the course of the recording are expressed as percentages of the respective baseline average in each group. This study was designed to make pairwise comparisons of saline and octopamine groups within each viral condition because of previously demonstrated effects of eGFP on long-lasting LTP induced by spaced four-train stimulation (Havekes et al., 2016b). Electrophysiology data were extracted using Clampfit 10 (Axon Molecular Devices) and Statistical analyses were performed using GraphPad Prism 9. Data were tested for normality and the maintenance of LTP was assessed by comparing the average fEPSP slopes from the last 20 min of the recording using two-tailed unpaired *t* tests. Input-output and PPF data were compared using two-way repeated measures ANOVA. For all analyses the statistical significance was set at *p *<* *0.05. In figures, *p < 0.05; **p <* *0.01.

## Results

**Figure 1.**
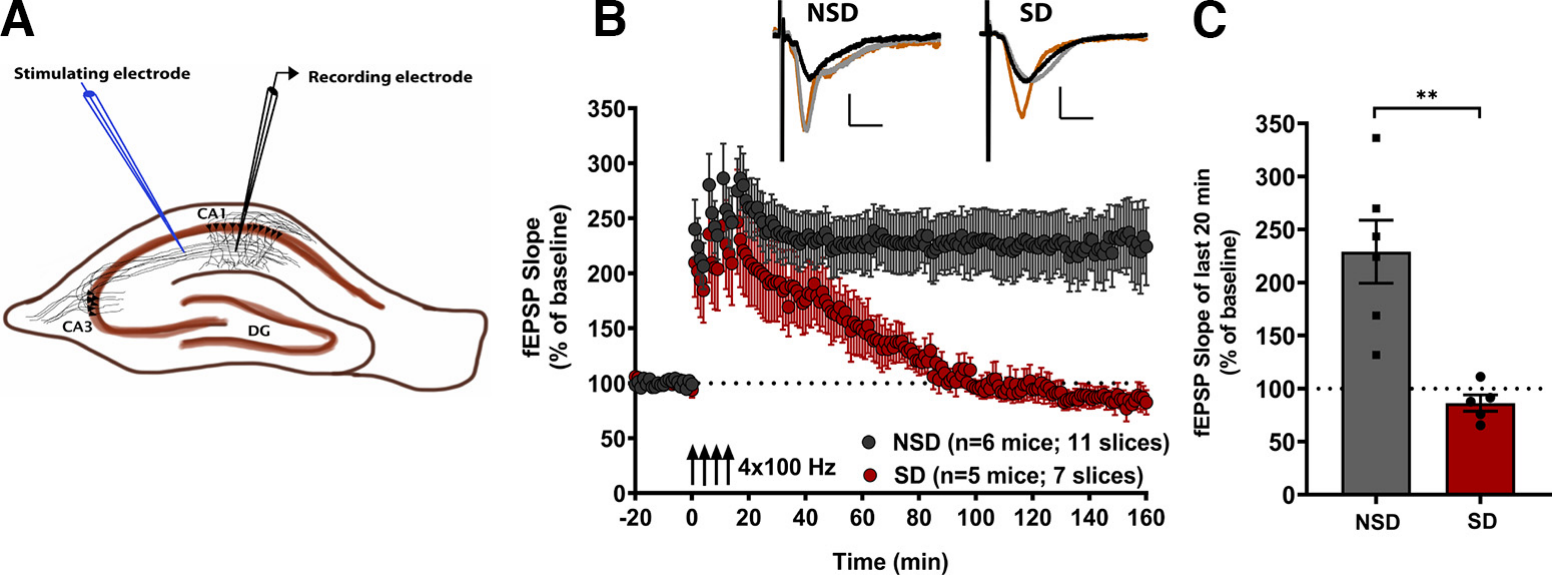
Brief sleep deprivation impairs the maintenance of cAMP-PKA signaling-dependent LTP in the hippocampal CA1 region. ***A***, Schematic representation of a transverse hippocampal slice showing the positioning of stimulating and recording electrodes in the CA1 stratum radiatum. ***B***, Long-lasting LTP induced by spaced four-train stimulation protocol (4 100 Hz, 1 s stimulation trains, spaced at 5 min) is impaired in slices from mice subjected to 5 h of sleep deprivation (SD) compared with nonsleep deprived (NSD) mice. The representative fEPSP traces shown for each group are sampled at baseline (black trace), 30 min after the first tetanus (orange trace), and at the end of the recording (gray trace). Scale bars for traces: 2 mV vertical, 5 ms horizontal. ***C***, Maintenance of LTP, assessed by comparing the mean potentiation over the last 20 min of the recording between the NSD group (229 ± 29.71%) and SD group (86.29 ± 7.72%), shows a significant deficit in the SD group (two-tailed *t* test, *t*_(9)_ = 4.250, *p* = 0.002, η^2^ = 0.6640). Data in ***B*** and ***C*** are shown as mean ± standard error of the mean (SEM).

We have previously shown that 5 h of SD by the gentle handling method leads to deficits in long-lasting LTP induced by spaced tetanic-train stimulation (Vecsey et al., 2009; Wong et al., 2019). In the first series of experiments we confirmed the impact of acute SD on spaced four-train LTP. Mice were either sleep deprived using gentle handling from ZT0 to ZT5 or allowed to sleep (non-sleep deprived; NSD) for the same duration. At ZT5, hippocampal slices were prepared for spaced four-train LTP induction and recordings at Schaffer collateral synapses in the CA1 stratum radiatum (Fig. 1*A*). The data showed clear deficits in the persistence of this long-lasting form of LTP in the SD group, where the potentiation decayed to baseline levels within 2 h, compared with stable long-lasting LTP in the NSD group (Fig. 1*B*). The maintenance of LTP was assessed by comparing the mean potentiation over the last 20 min of the recording between the two conditions. The mean potentiation of the NSD group (229 ± 29.71%) and the SD group (86.29 ± 7.72%) revealed a significant deficit in the SD group (Fig. 1*C*; two-tailed unpaired *t* test, *t*_(9)_ = 4.250, *p *=* *0.002, η^2^ = 0.664). These data confirm previous observations (Vecsey et al., 2009) that a brief period of SD leads to impairments in long-lasting LTP induced by spaced four-train stimulation.

**Figure 2.**
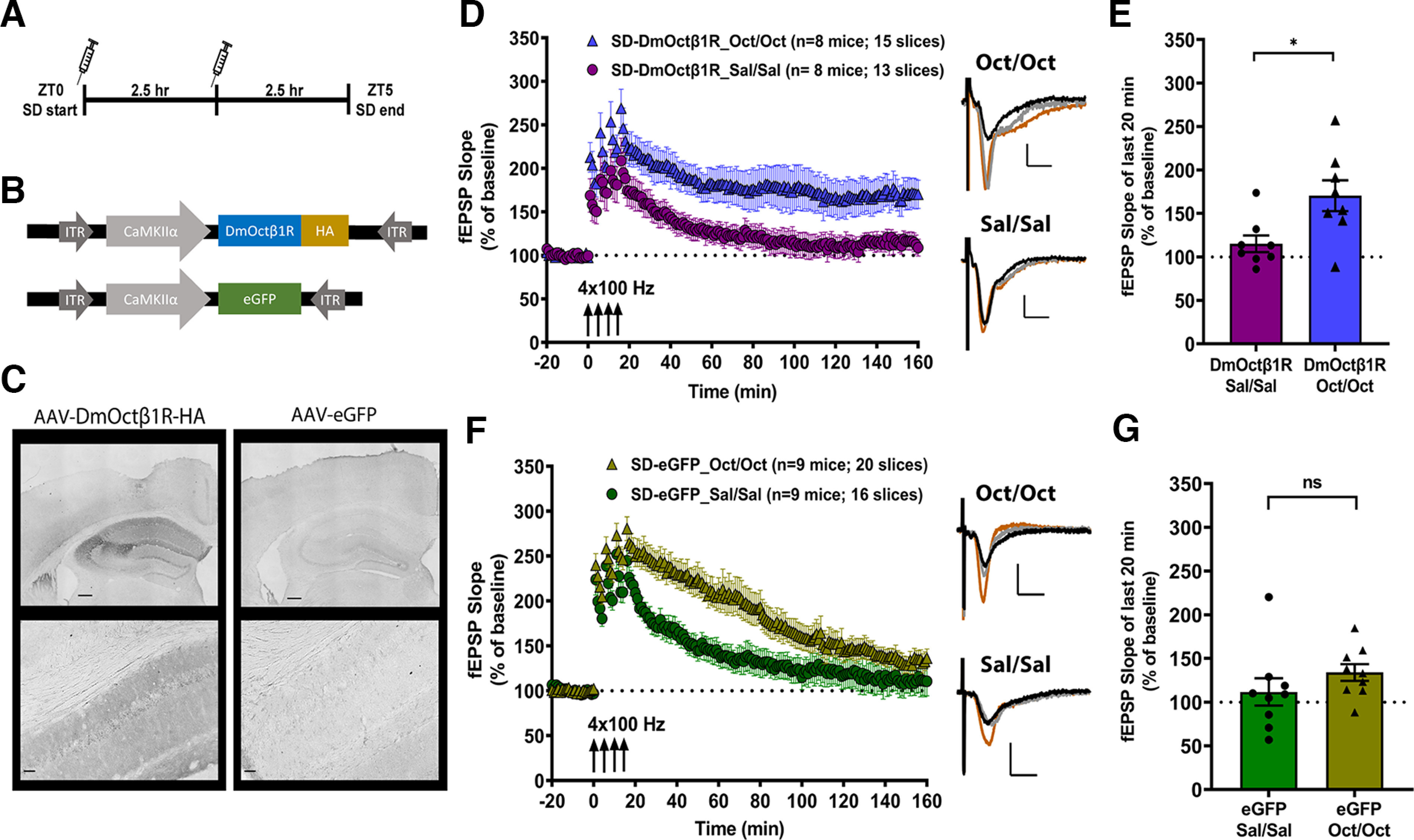
Chemogenetic enhancement of cAMP signaling during acute sleep deprivation confers resilience to the associated deficits in spaced four-train LTP. ***A***, The timeline of injections during the course of 5-h sleep deprivation (SD), starting at ZT0. Mice, expressing either DmOctβ1R or eGFP in the hippocampus, receive two injections of either the octopamine or saline at ZT0 and at ZT2.5. At the end of the 5-h SD, hippocampi are sliced and LTP induced by spaced four-train LTP is investigated. ***B***, Schematic representation of the AAV constructs used to drive the expression of *D. melanogaster* octopamine receptor (DmOctβ1R) or eGFP in hippocampal CaMKIIα-expressing neurons. ***C***, Representative images of coronal brain sections from mice expressing DmOctβ1R-HA, or control eGFP, which were probed by chromogenic DAB staining against the HA tag. The left panels show the restricted expression of DmOctβ1R in the hippocampal subregions marked by darker DAB stain, and the right panels show the lack of signal from the eGFP-expressing slice. Upper panel images for each AAV show the whole hippocampus (scale bar: 10 μm), and the lower panel images give a representative tile image (scale bar: 50 μm). ***D***, Following 5-h SD, mice expressing DmOctβ1R and receiving two injections of octopamine (Oct/Oct) show no deficits in the long-lasting spaced-four-train LTP whereas those receiving two injections of saline (Sal/Sal) show impairments. ***E***, Maintenance of LTP in the DmOctβ1R-expressing mice, assessed by comparing the mean potentiation over the last 20 min of recordings between the saline vehicle group (115 ± 9.64%) and octopamine group (170.5 ± 17.74%), shows significantly enhanced potentiation in the octopamine group (two-tailed unpaired *t* test, *t*_(14)_ = 2.746, *p = *0.016, η^2^ = 0.351). ***F***, In mice expressing eGFP, spaced four-train LTP persistence is impaired in both the octopamine (Oct/Oct) and saline (Sal/Sal)-injected conditions. ***G***, The mean potentiation over the last 20 min of recordings between the saline group (111.7 ± 15.61%) and octopamine group (134 ± 9.53%) showed no significant (ns) difference between the two groups (two-tailed unpaired *t* test, *t*_(16)_ = 1.219, *p = *0.24, η^2^ = 0.085). The representative fEPSP trace for each group shown in ***D***, ***F*** was sampled at the respective baseline (black trace), 30 min after the first tetanus (orange trace), and at the end of the recording (gray trace). Scale bars for traces: 2 mV vertical, 5 ms horizontal. Data in ***D***, ***E***, ***F***, ***G*** are shown as mean ± SEM. Basal synaptic transmission and paired-pulse facilitation for DmOctβ1R-expressing mice are shown in Extended Data Figure 2-1 and for eGFP-expressing mice in Extended Data Figure 2-2.

Next, we investigated the effect of chemogenetically enhancing cAMP levels in hippocampal neurons during the SD period using an approach that we established increases cAMP levels *in vivo* and which blocks the impact of SD on behavioral memory (Havekes et al., 2014). We virally expressed the Gαs-coupled DmOctβ1R or eGFP (Fig. 2*B*) in the hippocampal neurons of adult mice under the CaMKIIα promoter. We confirmed the expression of DmOctβ1R by DAB staining using an antibody against the HA tag on the receptor. The darker DAB label in slices from the DmOctβ1R-HA mice shows clear expression of the receptor in the hippocampus, compared with the slices from the eGFP-expressing mice which represent the level of background stain (Fig. 2*C*). Mice expressing either DmOctβ1R or eGFP were subjected to 5 h of SD from ZT0-ZT5. During SD, mice received two intraperitoneal injections of either octopamine (1 mg/kg), the ligand which binds and activates the DmOctβ1R, or saline vehicle. These injections were administered at the start (ZT0) and halfway through (ZT2.5) the SD period (Fig. 2*A*). This timing and dosage were based on our previous study (Havekes et al., 2014). At the end of the SD period, hippocampal slices were prepared and LTP was induced using the spaced four-train stimulation protocol. We then compared the maintenance of LTP between the octopamine and saline groups within each viral condition (Fig. 2*D*,*F*).

10.1523/ENEURO.0380-22.2022.f2-1 Extended Data Figure 2-1 Basal synaptic transmission and paired-pulse facilitation in mice virally expressing DmOctβ1R and receiving two injections of octopamine or saline during sleep deprivation. ***A***, Basal field-EPSP amplitudes are not significantly different between the saline group and the octopamine group (two-way repeated measures ANOVA; *F*_(1,14)_ = 0.202; *p *=* *0.660). ***B***, Presynaptic fiber volley (PFV) amplitudes are not significantly different between the saline group and the octopamine group (two-way repeated measures ANOVA; *F*_(1,14)_ = 2.113; *p *=* *0.168). ***C***, Paired-pulse facilitation over a range of interstimulus intervals is not significantly different between the saline group and the octopamine group (two-way repeated measures ANOVA; *F*_(1,13)_ = 0.825; *p *=* *0.380). Download Figure 2-1, TIF file.

10.1523/ENEURO.0380-22.2022.f2-2 Extended Data Figure 2-2 Basal synaptic transmission and paired-pulse facilitation in mice virally expressing eGFP and receiving octopamine or saline injections during sleep deprivation. ***A***, Basal field-EPSP amplitudes showed significant difference between the saline group and the octopamine group (two-way repeated measures ANOVA; *F*_(1,16)_ = 8.224; *p *=* *0.011). ***B***, Presynaptic fiber volley (PFV) amplitudes showed significant difference between the saline group and the octopamine group (two-way repeated measures ANOVA; *F*_(1,16)_ = 5.371; *p *=* *0.034). ***C***, Paired-pulse facilitation over a range of interstimulus intervals is not significantly different between the saline group and the octopamine group (two-way repeated measures ANOVA; *F*_(1,16)_ = 2.079; *p *=* *0.169). Download Figure 2-2, TIF file.

In SD mice expressing DmOctβ1R and receiving two injections of saline vehicle, LTP decayed to baseline levels within 2 h (Fig. 2*D*), similar to the noninjected wild-type SD mice in Figure 1*B*. In contrast, in SD mice expressing the DmOctβ1R that received two octopamine injections, LTP was maintained (Fig. 2*D*). The maintenance of LTP was evaluated by comparing the mean potentiation over the last 20 min of recordings between the DmOctβ1R saline group (115 ± 9.64%) and the DmOctβ1R octopamine group (170.5 ± 17.74%), and revealed significantly enhanced potentiation in the octopamine group (Fig. 2*E*; two-tailed unpaired *t* test, *t*_(14)_ = 2.746, *p *=* *0.016, η^2^ = 0.351). We also assessed basal synaptic transmission by comparing the input-output responses (stimulation intensity vs fEPSP or PFV amplitude) and paired-pulse facilitation (PPF), a very short-term form of plasticity, and found no significant differences between the two groups in any of these measures (Extended Data Fig. 2-1*A–C*). These results show that enhancing cAMP signaling in hippocampal neurons during SD confers resilience against the negative impact of brief SD on persistent synaptic plasticity.

To confirm that the resilience of LTP observed in the DmOctβ1R-expressing mice injected with octopamine was because of the activation of the heterologous receptor, we investigated the effect of two injections (at ZT0 and ZT2.5) of octopamine or saline during SD in mice with virally expressed eGFP in hippocampal neurons. eGFP-expressing SD mice showed deficits in the maintenance of LTP following spaced four-train LTP induction, regardless of whether they received octopamine or saline injections (Fig. 2*F*,*G*). The mean potentiation over the last 20 min of the recordings in the SD eGFP saline group (111.7 ± 15.61%) and SD eGFP octopamine group (134 ± 9.53%) showed no significant difference (Fig. 2*G*; two-tailed unpaired *t* test, *t*_(16)_ = 1.219, *p *=* *0.240, η^2^ = 0.085) in the maintenance of LTP between these two groups. Although we observed some differences in the time course of LTP decay between these groups, the maintenance of LTP, which is the hallmark of long-lasting synaptic plasticity (Frey et al., 1993), was impaired in both conditions. When we compared basal synaptic transmission measures, we observed some differences between the saline and octopamine SD eGFP groups in the fEPSP amplitudes (Extended Data Fig. 2-1*A*) and PFV amplitudes (Extended Data Fig. 2-2*B*), but no significant difference in PPF (Extended Data Fig. 2-2*C*). Overall, these findings demonstrate that the persistent LTP in slices from DmOctβ1R-expressing mice that received octopamine injections was because of effects on the heterologous Gαs-receptor that enhance intracellular cAMP levels.

**Figure 3.**
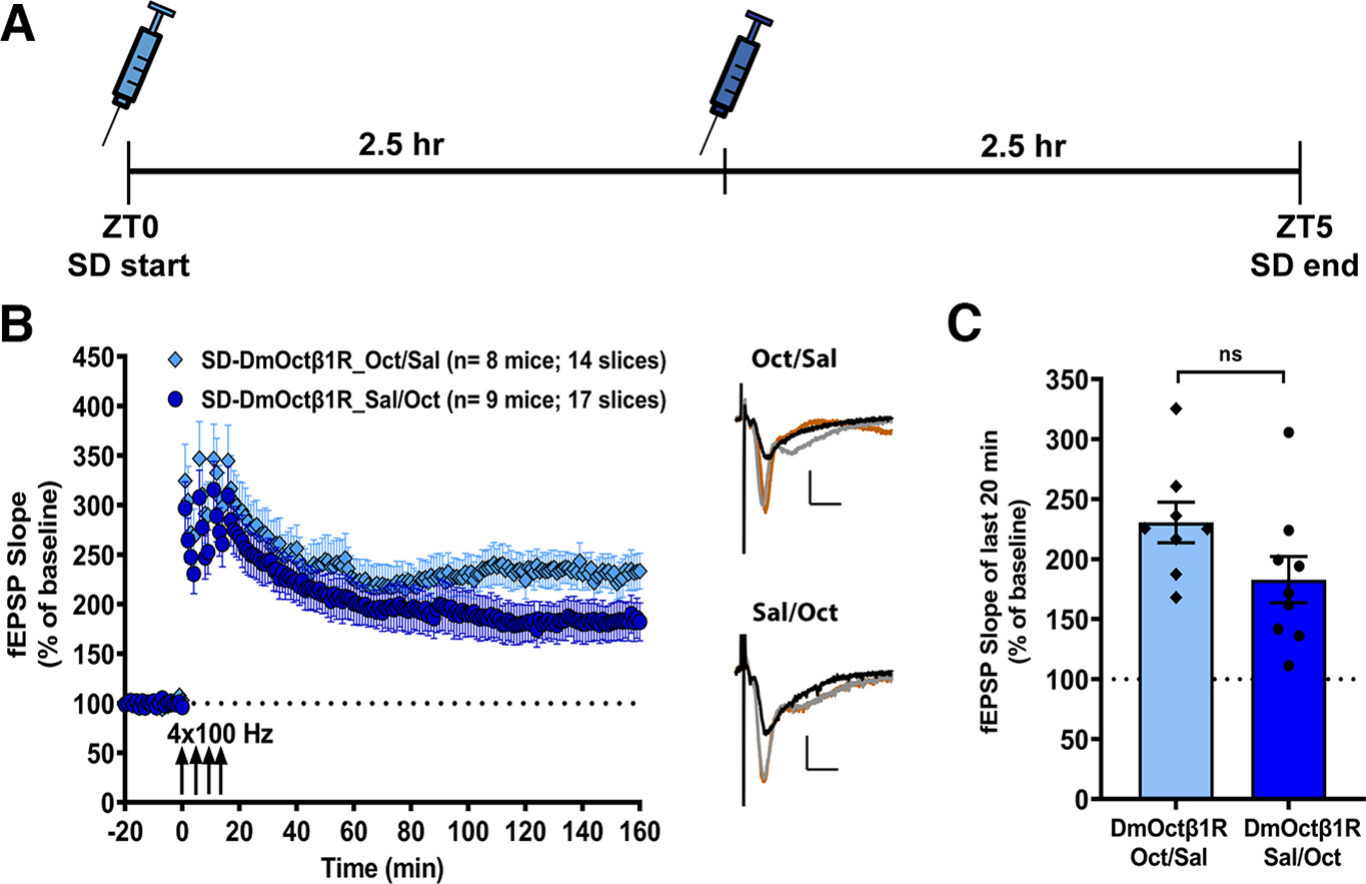
Enhancement of hippocampal cAMP signaling either in the first or second half of sleep deprivation prevents the associated deficits in long-lasting LTP. ***A***, A schematic representation of the timeline of injections during the course of 5-h sleep deprivation. Mice virally expressing DmOctβ1R in hippocampal neurons receive an injection of octopamine at either ZT0 or at ZT2.5. Mice that receive octopamine injection at ZT0 get a saline injection at ZT2.5 (Oct/Sal), and mice that get octopamine injection at ZT2.5 receive a saline injection at ZT0 (Sal/Oct). ***B***, A single injection of octopamine to chemogenetically enhance cAMP signaling either during the first or second half of 5-h SD is effective in preventing the impact of SD on spaced four-train LTP. The representative fEPSP traces shown for each group are sampled at baseline (black trace), 30 min after the first tetanus (orange trace), and at the end of the recording (gray trace). Scale bars for traces: 2 mV vertical, 5 ms horizontal. ***C***, The mean potentiation over the last 20 min of recordings between the Oct/Sal group (230.6 ± 16.87%) and the Sal/Oct group (182.8 ± 19.27%) shows no significant difference between the conditions, although the injection in the first window (Oct/Sal) trends toward being more effective (two-tailed unpaired *t* test, *t*_(15)_ = 1.844, *p = *0.085, η^2^ = 0.185). Data in ***B*** and ***C*** are shown as mean ± SEM. Basal synaptic transmission and paired-pulse facilitation are shown in Extended Data Figure 3-1.

Our findings (Fig. 2) demonstrate that chemogenetically enhancing cAMP levels during SD renders this long-lasting form of hippocampal LTP resilient to the impact of 5 h of acute SD. Other studies have shown that shorter windows of sleep deprivation can produce alterations in gene expression in the hippocampus (Delorme et al., 2021) and the cortex (Cirelli and Tononi, 2000) and impair memory and synaptic plasticity (Prince et al., 2014). These findings raise a question about whether the enhancement of cAMP levels could lead to LTP resilience if provided during one or the other halves of the SD period. To examine the possibility that a single injection of octopamine during either the first or second half of the 5-h SD period could prevent deficits in LTP, we performed another set of experiments using mice virally expressing DmOctβ1R in hippocampal neurons. The design was similar to the experiments described above, except mice in one group now received the injection of octopamine only at ZT0 and saline at ZT2.5 (Oct/Sal), whereas mice in the other group received saline at ZT0 and octopamine at ZT2.5 (Sal/Oct; Fig. 3*A*). At the end of SD, hippocampal slices were prepared for spaced four-train LTP recordings in the CA1 stratum radiatum. Interestingly, persistent LTP was observed for both of these conditions (Fig. 3*B*). Comparing the mean potentiation over the last 20 min of recordings, we found no significant difference between the Oct/Sal group (230.6 ± 16.87%) and the Sal/Oct group (182.8 ± 19.27%), although the octopamine injection in the first half (Oct/Sal) of SD appeared to trend toward being more effective (Fig. 3*C*; two-tailed unpaired *t* test, *t*_(15)_ = 1.844, *p *=* *0.085, η^2^ = 0.185). We also assessed the basal synaptic transmission and PPF and found no significant differences between the two groups in any of these measures (Extended Data Fig. 3-1*A–C*). These results suggest that activating cAMP signaling during either the first or second half of the SD period can prevent the decay of LTP induced by sleep loss, supporting the notion that the impact of SD on synaptic plasticity builds over time.

10.1523/ENEURO.0380-22.2022.f3-1 Extended Data Figure 3-1 Basal synaptic transmission and paired-pulse facilitation in mice virally expressing DmOctβ1R and receiving a single octopamine injection (either at ZT0 or at ZT2.5) during sleep deprivation. ***A***, Basal field-EPSP amplitudes are not significantly different between the saline group and the octopamine group (two-way repeated measures ANOVA; *F*_(1,15)_ = 0.628; *p *=* *0.440). ***B***, Presynaptic fiber volley (PFV) amplitudes are not significantly different between the saline group and the octopamine group (two-way repeated measures ANOVA; *F*_(1,15)_ = 0.026; *p *=* *0.874). ***C***, Paired-pulse facilitation over a range of interstimulus intervals is not significantly different between the saline group and the octopamine group (two-way repeated measures ANOVA; *F*_(1,15)_ = 0.715; *p *=* *0.411). Download Figure 3-1, TIF file.

## Discussion

There is growing evidence that the disruption of cAMP signaling is responsible for impairments in hippocampus-dependent processes following acute sleep loss (Vecsey et al., 2009; Havekes et al., 2014; Wong et al., 2019). Sleep deprivation causes a decrease in cAMP levels in the hippocampus (Vecsey et al., 2009), which may be driven by increased levels and activity of the phosphodiesterase PDE4A5 (Vecsey et al., 2009; Wong et al., 2019). PDE4A5 overexpression in hippocampal neurons mimics the memory and plasticity phenotype of SD (Havekes, et al., 2016a), and blockade of PDE4A5 activity by overexpression of a catalytically inactive form of PDE4A5 prevents the memory deficits that follow SD (Havekes et al., 2016b). There is also a reduction in the overall activity of protein kinase A (PKA) in SD, and thus a decrease in the phosphorylation of important downstream effectors (Wong et al., 2019). The present study was designed to investigate whether increasing cAMP levels in hippocampal neurons during SD is enough to protect long-lasting forms of hippocampal plasticity from these alterations in molecular signaling caused by SD.

Our data demonstrate that increasing cAMP levels during SD has a protective effect for synaptic plasticity. We found that activating the heterologous Gαs-coupled DmOctβ1 receptor with the ligand octopamine prevented the decay of spaced four-train LTP, which is a form of long-lasting LTP known to be dependent on cAMP-PKA signaling, transcription, and translation (Frey et al., 1988, 1993; Huang and Kandel, 1994; Nguyen et al., 1994; Abel et al., 1997; Malleret et al., 2001). Spaced four-train LTP is vulnerable to acute SD, as was reported previously (Vecsey et al., 2009), and confirmed in our experiments. Mice without chemogenetic elevation of hippocampal cAMP (either eGFP-expressing conditions, or DmOctβ1R with saline) show impaired maintenance of long-lasting LTP induced by spaced four-train stimulation, suggesting that the resilience we observed was mediated by the activation of the heterologous receptor and not because of an off-target effect of octopamine.

Although the eGFP-expressing mice that received two octopamine injections showed deficits in the maintenance of LTP following SD, the rate of LTP decay showed some differences compared with eGFP-expressing mice receiving saline (Fig. 2*F*). This suggests a possible effect of octopamine that is not mediated by the heterologous receptor, although importantly this effect is not seen on long-lasting plasticity maintenance, measured 2 h after LTP induction (Fig. 2*G*). An explanation for the effects of octopamine observed in our eGFP-expressing mice could be that octopamine competes for binding to other neurotransmitter receptors or transporters, thereby altering levels of these endogenous transmitters or their signaling (Berry, 2004; Zhang et al., 2004; Zucchi et al., 2006; Kleinau et al., 2011). It has been demonstrated that the activation of trace amino acid receptors (TAARs), to which octopamine could bind, can alter the activity of the dopamine transporter (Xie and Miller, 2007; Revel et al., 2011; Underhill et al., 2021) in cells where TAARs and dopamine receptors colocalize (Xie and Miller, 2007; Miller, 2011). However, TAARs are not highly expressed in the hippocampus (Borowsky et al., 2001; Lindemann et al., 2008) and high concentrations of octopamine are required to activate TAARs (Zucchi et al., 2006), making it unlikely that octopamine administration would alter transporter activity in our experiments. Additionally, the literature suggests that octopamine is not a strong direct competitor for other neurotransmitter transporters (Pörzgen et al., 2001). It has also been shown that the efficiency and potency of octopamine’s action on other neurotransmitter receptors is markedly low (Berry, 2004; Zhang et al., 2004), making it unlikely that nonspecific binding would produce these changes in our experiments. Our previous publication using this DmOctβ1R system showed no effect of octopamine on memory performance in nonreceptor-expressing mice (Havekes et al., 2014), and work in a similar receptor system has demonstrated that octopamine did not alter LTP in the absence of the octopamine receptor (Isiegas et al., 2008). Together, these observations suggest that octopamine is not producing a protective effect on LTP through off-target mechanisms.

Our results with the single injection of octopamine either in the first or second half of the SD period show that LTP is made resilient by elevated cAMP levels regardless of the time point within the course of SD that it occurred. This is interesting, because evidence suggests that the effects of acute SD build over time (Marks and Wayner, 2005). Three hours of SD cause mild impairments in LTP in the dentate gyrus of the hippocampus, but effects are more severe with 6-h SD (Marks and Wayner, 2005). It is possible that the injections in the first and second half of SD are both effective because either injection prevents cAMP levels from falling enough to disrupt LTP. It is important to note that the full time course of cAMP degradation during SD remains unknown, and future studies will need to examine this, perhaps using an *in vivo* imaging approach.

This work provides important insights on the hippocampal mechanisms that can be altered to provide resilience to the impact of acute sleep loss. A primary goal for future work will be mapping the changes in cAMP and signaling partners across the period of sleep deprivation. Previously, we have shown increased activity and levels of the cAMP phosphodiesterase PDE4A in the hippocampus following sleep deprivation (Vecsey et al., 2009). In future studies, we will investigate whether altering cAMP levels through the octopamine strategy might also change the activity of PDEs in the hippocampus, or of other upstream or downstream effectors. It would also be interesting to investigate whether heterologous Gαs activation confers resilience to other forms of synaptic plasticity, such as lasting LTP induced by theta-burst stimulation, potentiation induced by forskolin, and synaptic tagging, which are all impacted by brief SD (Vecsey et al., 2009, 2018). Greater understanding of this central mechanism can also point to possible strategies for intervention in diseases with sleep loss, by detailing the mechanisms necessary to prevent the decline in cognitive processing that accompany it.

## Synthesis

Reviewing Editor: Arvind Kumar, KTH Royal Institute of Technology Decisions are customarily a result of the Reviewing Editor and the peer reviewers coming together and discussing their recommendations until a consensus is reached. When revisions are invited, a fact-based synthesis statement explaining their decision and outlining what is needed to prepare a revision will be listed below. The following reviewer(s) agreed to reveal their identity: Thomas O’Dell, Sreedharan Sajikumar. Note: If this manuscript was transferred from JNeurosci and a decision was made to accept the manuscript without peer review, a brief statement to this effect will instead be what is listed below.

Synthesis

Your manuscript was reviewed by two reviewers. We were lucky to secure one of the reviewers who reviewed your manuscript at J.Neurosci. So we did not follow the double blind review process in this case.

The two reviewers are more or less in agreement that you have addressed a relevant question and addressed it adequately. The concerns of one of the previous reviewers have been addressed. Since we had to get a new reviewer, the new reviewer has a few concerns which we think should be addressed in a new revision of the manuscript. I have appended their comments of the reviewer below for you to consider when revising the manuscript.

Comments from an additional review

The manuscript by Walsh et al., demonstrates that cell-type specific and temporally regulated increases in cAMP signaling during sleep deprivation prevent deficits in long-lasting forms of synaptic plasticity. The authors have studied whether or not elevating cAMP can compensate for molecular signaling deficits during sleep deprivation. This work is a nice extension of their previously published behavioral work (Havekes et al., 2014) to the cellular level.

I understand that the manuscript was reviewed by another journal and the authors addressed most of the comments/questions which are satisfactory. I have only a few suggestions below for improvement.

1) The PDE4 isoform PDE4A5 was significantly upregulated by sleep deprivation in the hippocampus (Vecsey etl., 2009, Wong et al., 2019). In the current work it is not clear whether increased cAMP level may be able to reduce the PDE4 levels?

2) The electrophysiology recordings are of high quality but most of the fEPSP traces are contaminated with population spikes. In the case of Fig.1B SD group, the fEPSP traces are normal but this is the last trace and is difficult to evaluate whether a similar kind of spike contamination happened immediately after tetanization. It will be helpful if the authors can show +30 min fEPSP traces in the current figures.

3) The authors did not record synaptic potentials from a control input. How do the authors make sure that the excitability of the cells are not changed over the long recording period? Did the authors evaluate the I/O curve after the experiment?

## Author Response

"Chemogenetic enhancement of cAMP signaling renders hippocampal synaptic plasticity resilient to the impact of acute sleep deprivation"

Synthesis of Reviews:

Computational Neuroscience Model Code Accessibility Comments for Author (Required):

This is an experimental paper and not much of computational methods are involved.

Synthesis Statement for Author (Required):

Your manuscript was reviewed by two reviewers. We were lucky to secure one of the reviewers who reviewed your manuscript at J.Neurosci. So, we did not follow the double-blind review process in this case.

The two reviewers are more or less in agreement that you have addressed a relevant question and addressed it adequately. The concerns of one of the previous reviewers have been addressed. Since we had to get a new reviewer, the new reviewer has a few concerns which we think should be addressed in a new revision of the manuscript. I have appended their comments of the reviewer below for you to consider when revising the manuscript.

Point-by-point response to comments from an additional review:

The manuscript by Walsh et al., demonstrates that cell-type specific and temporally regulated increases in cAMP signaling during sleep deprivation prevent deficits in long-lasting forms of synaptic plasticity. The authors have studied whether or not elevating cAMP can compensate for molecular signaling deficits during sleep deprivation. This work is a nice extension of their previously published behavioral work (Havekes et al., 2014) to the cellular level.

I understand that the manuscript was reviewed by another journal and the authors addressed most of the comments/questions which are satisfactory. I have only a few suggestions below for improvement.

1) The PDE4 isoform PDE4A5 was significantly upregulated by sleep deprivation in the hippocampus (Vecsey etl., 2009, Wong et al., 2019). In the current work it is not clear whether increased cAMP level may be able to reduce the PDE4 levels?

We thank the reviewer for this suggestion. In the current work, we have not evaluated whether chemogenetic enhancement of cAMP levels affected the PDE4 levels. We will investigate this in our future experiments. We have now briefly mentioned this in the discussion section of the revised manuscript (highlighted in red).

2) The electrophysiology recordings are of high quality but most of the fEPSP traces are contaminated with population spikes. In the case of Fig.1B SD group, the fEPSP traces are normal but this is the last trace and is difficult to evaluate whether a similar kind of spike contamination happened immediately after tetanization. It will be helpful if the authors can show +30 min fEPSP traces in the current figures.

We do not see that any of our baseline fEPSP traces are contaminated with population spike component. If the reviewer is referring to the traces after the tetanization, we think that it is not unusual to see the spike component because tetanization would drive neuronal firing. Nevertheless, it is important to note that for all our analyses, we have used the initial slope (20-80%) of the fEPSP which is devoid of any population spike component.

As the reviewer has requested, we have now included the +30 min fEPSP traces for all the conditions in the revised figures as shown below.

In all the figures, the representative fEPSP traces shown include one at baseline (black trace), one at 30 min after the first tetanus train (orange trace) and one at the end of the recording period (grey trace).

3) The authors did not record synaptic potentials from a control input. How do the authors make sure that the excitability of the cells are not changed over the long recording period? Did the authors evaluate the I/O curve after the experiment?

We have not recorded this primarily due to the limitations in our recording setup. Because in all our experiments, we were recording from two experimental conditions side-by-side on the same rig, we were limited by the number of stimulation inputs. We have been performing similar long-term recordings in acute slices for many years and we have not observed significant changes in excitability over the course of ∼3h recording period in slices from WT/control animals. As a more relevant example, in one of our recent experiments from another study shown below (Shetty et al. 2022. Cerebral Cortex; PMID: 34937090), we did record a simultaneous control input and it showed no fluctuations over the course of entire recording period.

We have not evaluated I/O curve at the end of the recording in any of our experiments.
