# Supplementary figures and images for: Chemogenetic Enhancement of cAMP Signaling Renders Hippocampal Synaptic Plasticity Resilient to the Impact of Acute Sleep Deprivation
Source: eNeuro. 2023 Jan 3;10(1):ENEURO.0380-22.2022. doi: 10.1523/ENEURO.0380-22.2022 (PMC9829098; doi:10.1523/ENEURO.0380-22.2022)

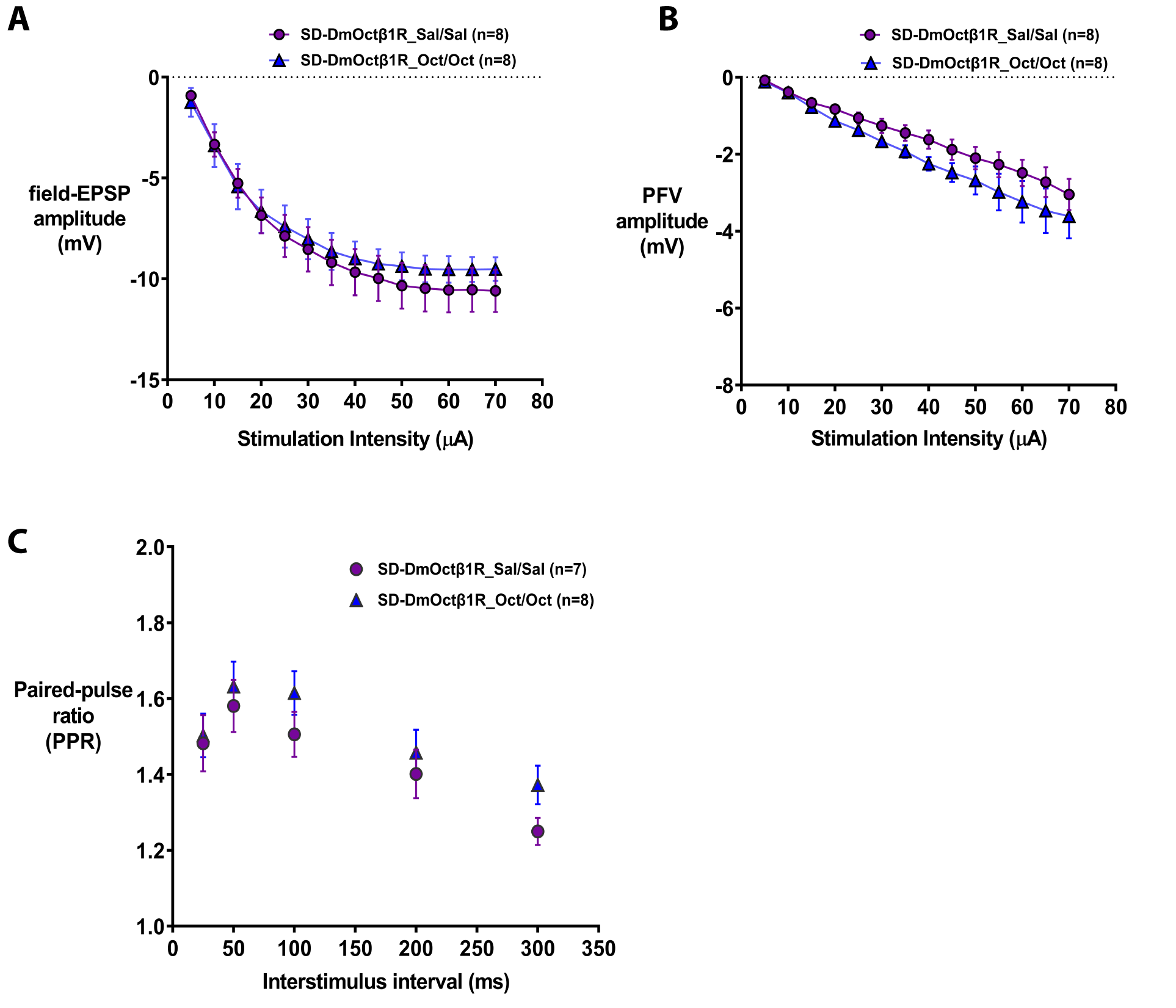

Supplement: Extended Data Figure 2-1 — Basal synaptic transmission and paired-pulse facilitation in mice virally expressing DmOctβ1R and receiving two injections of octopamine or saline during sleep deprivation. A, Basal field-EPSP amplitudes are not significantly different between the saline group and the octopamine group (two-way repeated measures ANOVA; F(1,14) = 0.202; p = 0.660). B, Presynaptic fiber volley (PFV) amplitudes are not significantly different between the saline group and the octopamine group (two-way repeated measures ANOVA; F(1,14) = 2.113; p = 0.168). C, Paired-pulse facilitation over a range of interstimulus intervals is not significantly different between the saline group and the octopamine group (two-way repeated measures ANOVA; F(1,13) = 0.825; p = 0.380). Download Figure 2-1, TIF file. [file enu-eN-TNWR-0380-22-s01.tif]

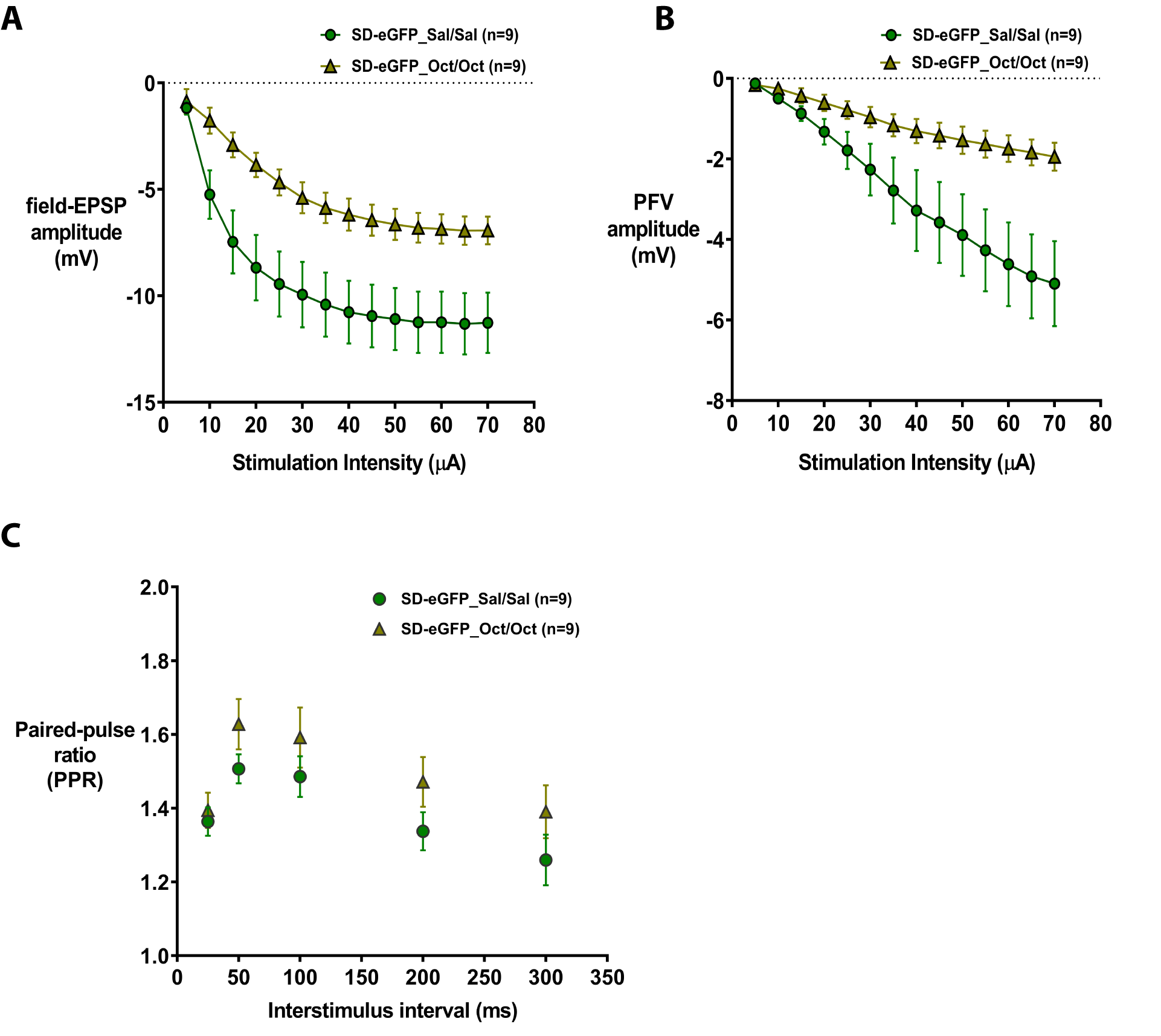

Supplement: Extended Data Figure 2-2 — Basal synaptic transmission and paired-pulse facilitation in mice virally expressing eGFP and receiving octopamine or saline injections during sleep deprivation. A, Basal field-EPSP amplitudes showed significant difference between the saline group and the octopamine group (two-way repeated measures ANOVA; F(1,16) = 8.224; p = 0.011). B, Presynaptic fiber volley (PFV) amplitudes showed significant difference between the saline group and the octopamine group (two-way repeated measures ANOVA; F(1,16) = 5.371; p = 0.034). C, Paired-pulse facilitation over a range of interstimulus intervals is not significantly different between the saline group and the octopamine group (two-way repeated measures ANOVA; F(1,16) = 2.079; p = 0.169). Download Figure 2-2, TIF file. [file enu-eN-TNWR-0380-22-s02.tif]

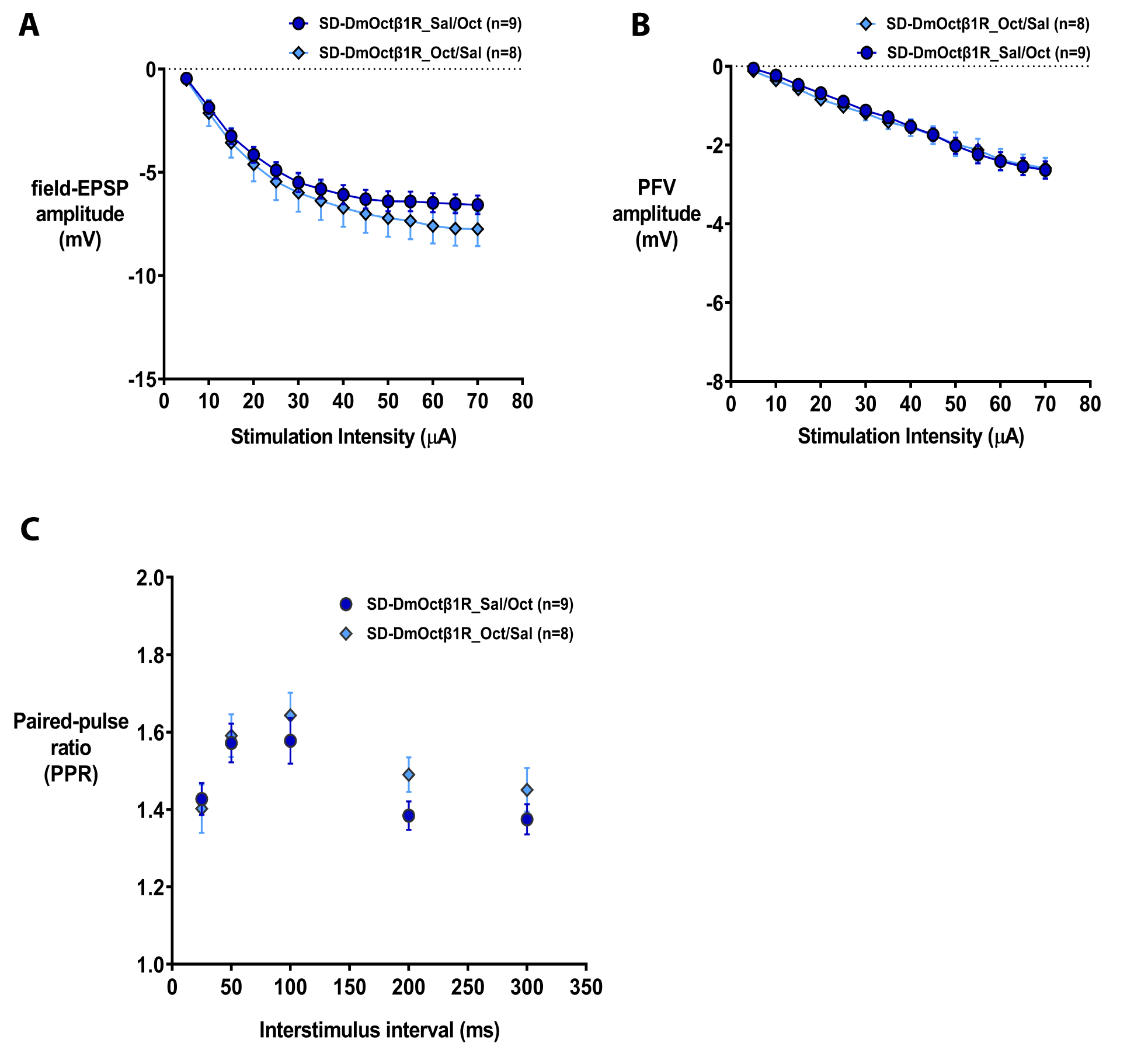

Supplement: Extended Data Figure 3-1 — Basal synaptic transmission and paired-pulse facilitation in mice virally expressing DmOctβ1R and receiving a single octopamine injection (either at ZT0 or at ZT2.5) during sleep deprivation. A, Basal field-EPSP amplitudes are not significantly different between the saline group and the octopamine group (two-way repeated measures ANOVA; F(1,15) = 0.628; p = 0.440). B, Presynaptic fiber volley (PFV) amplitudes are not significantly different between the saline group and the octopamine group (two-way repeated measures ANOVA; F(1,15) = 0.026; p = 0.874). C, Paired-pulse facilitation over a range of interstimulus intervals is not significantly different between the saline group and the octopamine group (two-way repeated measures ANOVA; F(1,15) = 0.715; p = 0.411). Download Figure 3-1, TIF file. [file enu-eN-TNWR-0380-22-s03.tif]
